# Descriptors for Predicting Single- and Multi-Phase Formation in High-Entropy Oxides: A Unified Framework Approach

**DOI:** 10.3390/ma18163862

**Published:** 2025-08-18

**Authors:** Alejandro F. Manchón-Gordón, Paula Panadero-Medianero, Javier S. Blázquez

**Affiliations:** 1 Instituto de Ciencia de Materiales de Sevilla, CSIC-Universidad de Sevilla, C. Américo Vespucio 49, 41092 Sevilla, Spain; 2 Departamento de Física de la Materia Condensada, ICMSE-CSIC, Universidad de Sevilla, P.O. Box 1065, 41080 Sevilla, Spainjsebas@us.es (J.S.B.)

**Keywords:** high-entropy oxides, stability predictors, atomic size misfit, configurational entropy

## Abstract

High-entropy oxides, HEOs, represent a relatively new class of ceramic materials characterized by the incorporation of multiple cations, typically four or more, into a single-phase crystal structure. This extensive compositional flexibility allows for the introduction of specific chemical elements into a crystal lattice that would normally be unable to accommodate them, making it difficult to predict a priori their properties and crystal structures. Consequently, studying the phase stability of these single-phase materials presents significant challenges. This work examines the key parameters commonly employed to predict the stabilization of HEOs and introduces a unified framework for analyzing their stability. The proposed approach incorporates a normalized configurational entropy per mole of atoms and the relative volume occupied by cations into the mean atomic size deviation. By combining these parameters, the approach enables, as a first approximation, the identification of compositional ranges that favor the formation of single-phase and multi-phase HEO compounds with rock salt, spinel, fluorite, pyrochlore, and perovskite structures.

## 1. Introduction

Since the innovative development and creation of new alloys in 2004 [1], which are based on the strategic mixing of several elements in equal proportions, the concept of high-entropy alloys has gained significant attention in the field of materials science [2]. High-entropy alloys, HEAs, are solid solutions consisting of five or more different chemical elements that exhibit a wide range of mutual solubility. The term HEAs originates from the high configurational entropy estimated directly for their composition, using the following expression:

(1) $$∆S_{conf}=-R\sum x_{i}\ln x_{i},$$

where $x_{i}$ is the atomic fraction of the i-th element and R is the universal gas constant. For a quinary equiatomic alloy (i.e., five elements with equal concentrations), the configuration entropy reaches $∆S_{conf}$ = 1.6R = 13.4 JK^−1^ mol^−1^. A commonly accepted threshold of $∆S_{conf}>1.5R$ is used to define HEAs, which allows for slight deviations from perfect equiatomic compositions. This threshold can be further relaxed to include quinary alloys with component concentrations in the range 0.05 < $x_{i}$ < 0.35, which proved that the total configurational entropy remains above $∆S_{conf}\;\geq$ 1.36R.

In 2015, the high-entropy concept was expanded to include oxides when Rost et al. [3] successfully synthesized a complex oxide system containing five elements: Co, Cu, Mg, Ni, and Zn. This pioneering study showed that it is possible to stabilize an HEO system through entropy, resulting in the formation of a single-phase rock salt structure. Following Rost’s pioneering efforts, a diverse range of high-entropy ceramic classes, including borides, nitrides, sulfides, aluminates, alumino-silicides, tungstate, or oxisulfides, have been explored [4]. HEOs have garnered significant interest within the scientific community as they open up a vast and unexplored materials space. This often leads to the emergence of novel functional properties. An example is the exceptional cycling stability of rock salt HEOs when used as anodes in Li-ion batteries [5]. Additional functional traits of HEOs include their low thermal conductivity [6] and high catalytic activity [7]. Since its initial proposal, this research field has undergone substantial development, becoming a true milestone in the study of oxide materials. The number of peer-reviewed publications has steadily and significantly increased, reaching nearly 10,000 articles in 2024 (data retrieved from Web of Science).

The formation of single-phase structures in HEOs is often hastily attributed to configurational entropy. This has led to widespread misuse of the terms “entropy-stabilized oxides” and “high-entropy oxides”, which are frequently, but inaccurately, used interchangeably [8]. In reality, the stabilizing effect of entropy must be demonstrated on a case-by-case basis. Even for the prototypical HEO, (Mg,Co,Ni,Cu,Zn)O, the exact role of entropy in phase stabilization remains a subject of ongoing debate [9], as highlighted in several excellent reviews [8,10,11]. Although significant progress has been made in correlating experimental characterization with atomic configurations in HEAs, understanding and controlling local order and disorder in HEOs remains a major challenge. The degree to which disorder contains elements of short-range order, and the spatial extent of such phenomena, still requires thorough investigation [12,13,14].

In contrast to metals and alloys, which generally form simple crystal structures like face-centered cubic, FCC, body-centered cubic, BCC, or hexagonal close-packed, HCP, structures, oxides typically possess much more intricate crystal structures. The significant compositional complexity and expansive phase space characteristic of HEOs pose major challenges for traditional experimental approaches, making systematic investigation difficult. Consequently, employing descriptors has become an effective strategy for phase prediction. However, many of these descriptors were initially designed for solid solutions and have been applied to HEOs with little modification. Although these metrics offer ease of computation and well-established physical meaning, they frequently fail to accurately reflect the distinctive characteristics of high-entropy systems, especially the complex interactions among multiple elements and the stabilization mechanism driven by entropy. Consequently, composite descriptors, derived from multiple interrelated parameters, have shown improved accuracy and consistency in forecasting both the likelihood of successful synthesis and the resulting properties of the material [15]. As a result, when extending the high-entropy concept to oxides, the conventional configurational entropy calculations used for HEAs are not directly applicable. On the other hand, machine learning has emerged as a powerful tool in the field of high-entropy materials. Various algorithms, including both traditional methods and deep neural networks, have been applied in HEO research [16,17,18]. Several high-quality reviews summarizing recent advances in this area can be found in the literature [19,20].

This work presents a review of the key descriptors involved in phase stabilization within HEO systems and introduces a unified framework approach for studying their structural stability. To achieve this, modified expressions for configurational entropy and atomic size misfit are proposed, enabling a systematic and comparative analysis of single-phase stability ranges across different HEO crystal structures. The proposed methodology allows for a consistent, structure-independent representation.

## 2. Structures and Compositions

HEOs can be categorized into two main types based on the nature of their non-oxygen sublattices: simple and complex oxides. Simple oxides feature a single cation sublattice, where one type of cation occupies a single Wyckoff site. Common structural types in this category include rock salt and fluorite. Complex oxides possess multiple independent cation sublattices, with different cations residing on two or more separate Wyckoff positions. Typical examples of such structures are spinel, pyrochlore, and perovskite.

### 2.1. High-Entropy Oxides with the Rock Salt Structure, R-HEOs

Within the diverse categories of HEOs, those featuring the rock salt structure stand out as the earliest reported and most extensively studied. Rost et al. [3] conducted a pivotal study demonstrating the formation of a singular-phase quinary HEO, (Mg,Co,Cu,Ni,Zn)O, adopting the rock salt structure. This was achieved by combining five binary oxides (MgO, CoO, CuO, NiO, and ZnO) in equimolar proportions and subjecting the mixture to heating within the 1123–1173 K temperature range. These properties observed in those compounds include (1) a reversible solid-state transformation between a multi-phase state and a single-phase structure; (2) the development of a crystal structure that differs from at least two of the original constituent compounds; and (3) a positive enthalpy of formation [9].

The majority of HEOs featuring the rock salt structure are based on the Mg-Co-Ni-Cu-Zn cations system. Notably, theoretical calculations utilizing entropy and enthalpy descriptors and considering various M^2+^ cations (such as Ca, Co, Cu, Fe, Mg, Mn, Ni, and Zn) have identified the (Cu,Co,Ni,Mg,Zn)O system as the most favorable for forming a high-entropy material with a single-phase rock salt structure [21]. These calculations reveal that systems containing Ca, Fe, and Mn show significantly high and unfavorable enthalpy contributions that are considerably large and challenging to offset by configuration entropy, especially at feasible temperatures. These factors make it particularly challenging to extend the original composition to incorporate other 3D transition metals in the rock salt structure. Nevertheless, the (Mg,Mn,Fe,Co,Ni)O composition, which includes divalent Mn and Fe, was successfully produced through a sophisticated bottom-up approach. In this system, the different metal ions randomly occupy cation sites, while the oxygen sublattice allows for accommodating differences in cation sizes [22].

Moreover, a charge compensation mechanism was observed to occur alongside the complete or partial oxidation of certain elements in different R-HEO compounds. For example, Bérardan et al. [23] demonstrated that the Co^+3^ content increased with the amount of lithium in the Li_1 − x_(Mg,Ni,Co,Cu,Zn)_x_ O system. In the (Co,Cu,Mg,Na,Ni,Zn)O R-HEO, charge compensation was also detected by the formation of Co^+3^ to offset the +1 oxidation state of Na. Importantly, efforts to synthesize a single-phase rock salt structure with cations having stable +3 oxidation states in equal ratios were unsuccessful in the Co,Cu,Mg,Ni,Zn)O compound [24] and Co-Cu-Fe-Mg-Mn-Ni-O system [25]. Similar results were found when +3 cations such as Ga^3+^ were introduced, resulting in the formation of multi-phase systems. A range of different dopants was also investigated, including Li^+^, In^3+^, Ga^3+^, and Ti^4+^ ions. In general, achieving stable, single-phase materials proved to be challenging when incorporating dopants with higher valence states. Furthermore, among oxides, there is a competition between rock salt and spinel structures, with spinel providing greater flexibility to incorporate different cations into two separate sublattices [26,27].

These results show the significance of maintaining electroneutrality in the structure for the formation of a single phase and suggest the need for multivalent cations to accommodate cations of varied ionic radii. Therefore, Hume-Rothery and Pauling’s rules regarding size limitations and the same oxidation states of cations are not necessarily essential criteria for the formation of a single-phase structure. Recently, the phase stability as a function of temperature was investigated for two-, three-, and four-component oxides, using NiO, CoO, or MgO as the primary rock salt matrix. These were compared to the prototypical (Co,Cu,Mg,Ni,Zn)O R-HEO [28]. The thermodynamic model approach, assuming configuration entropy as the primary driver of phase stabilization, counterbalancing unfavourable enthalpy contributions, was proved too simplistic to capture the full complexity of the system. Notably, the study revealed that copper played the most critical role in stabilizing the structure, highlighting the need for a more nuanced understanding of elemental contributions.

### 2.2. High-Entropy Oxides with the Spinel Structure, S-HEOs

The cubic spinel structure is characterized by the general formula AB_2_O_4_, which consists of two distinct crystallographic cationic sites, one tetrahedral and one octahedral, along with a designated position for oxygen atoms. Typically, divalent cations occupy the tetrahedral sites, while trivalent cations occupy the octahedral sites. However, cation inversion is relatively common, with divalent 2+ cations occupying octahedral sites and trivalent 3+ cations occupying tetrahedral sites, contrary to the ideal configuration. Additionally, some spinels can deviate from their ideal stoichiometric composition, resulting in cation site vacancies along with corresponding oxygen vacancies to maintain charge balance. The existence of these crystallographic disorders contributes to the overall configuration entropy of the material.

The system (Cr,Mn,Fe,Co,Ni)_3_O_4_, composed entirely of transition metals, was the first reported single-phase S-HEO. This breakthrough paved the way for the development of numerous other S-HEO compositions [29]. Subsequently, the compositional space was expanded to include AB2O4-type structures, where the A-site contains equal amounts of Mg, Mn, Fe, Co, Ni, Cu, and Zn, and the B-site is occupied by either Cr or Fe [30]. The preparation of high-entropy aluminate spinel oxides (B *=* Al) has also been examined [31]. Additionally, variations such as (X)_3_O_4_, where Co or Ni in the original compound is replaced by Mg, have been explored [32]. The inclusion of non-transition Al element in (Co,Cr,Fe,Mn,Ni)_3_O_4_ replacing Co, Cr, Fe, Mn, and Ni elements has also been investigated [33,34]. Additionally, nanocrystals with a single-phase spinel structure have been synthesized for a six-component HEO, (Mg,Zn,Mn,Co,Ni,Fe)_3_O_4_, using the sol–gel combustion method [35]. The magnetic properties of the single spinel composition, (Cu,Ni,Ti,Zn,Fe)_3_O_4_, have also been studied and characterized [36]. Furthermore, within the Co-Cr-Fe-Mg-Mn-Ni-O system, one senary and six quinary compositions were synthesized using the solid-state reaction method. Among these, four compositions exhibited a mixture of rock salt and spinel-structured phases. In contrast, the remaining three compositions (Co,Cr,Fe,Mn,Ni)_3_O_4_, (Co,Cr,Fe,Mg,Mn)_3_O_4_, and (Cr,Fe,Mg,Mn,Ni)_3_O_4_ were obtained as single-phase spinel structures [32].

Although increasing the number of components is often used to enhance the stability of high-entropy compounds, it may not always be the optimal strategy. The arrangement of cations within various sublattices can significantly influence the energy landscape and structure, especially when dealing with multiple cation sublattices. Although equimolar composition is the easiest way to maximize entropy in a simple rock salt structure, the more complex spinel lattice imposes stricter constraints [37]. As a result, the tetrahedral and octahedral sublattices can be considered structurally independent, offering a wide range of tuning options and laying the groundwork for effectively modulating the properties of S-HEOs. In fact, the introduction of diverse cations on the available crystallographic sites leads to intricate distributions, potentially resulting in partial or complete inversion [30,32,38]. Sarkar et al. [39] found that, in the S-HEO (Co,Cr,Fe,Mn,Ni)_3_O_4_, the cations are selectively arranged rather than randomly distributed between the octahedral and tetrahedral sites. Instead, they are arranged in a manner that minimizes the configuration entropy permitted by the composition within the constraints of the spinel structure. This indicates that the concept of entropy stabilization in spinels predates the modern framework of HEOs. Supporting this, Navrotsky et al. [40,41,42] showed that in binary spinel systems like MgAl_2_O_4_, cation site mixing can produce enough configurational entropy to stabilize the spinel phase, even though the individual binary oxides are favored based on enthalpy.

### 2.3. High-Entropy Oxides with the Perovskite Structure, P-HEOs

The ABO_3_ perovskite structure comprises B-type cations with 6-fold coordination, A-type cations with 12-fold coordination, and octahedral oxygen anions. Unlike spinel structures, where cation distribution across A and B sites is primarily dictated by valence states, the arrangement of cation sublattices in perovskites is primarily influenced by the ionic radii. In perovskites, larger cations, such as rare-earth, RE, elements, typically occupy the A-sites, while smaller cations, often transition metals, occupy the B-sites. Crystallographic studies of P-HEOs emphasize a strategy of synergistic multi-cation occupancy of Wyckoff positions across the A-, B-, and mixed A/B-sites to achieve structural stability and compositional complexity.

The initial exploration of P-HEOs was documented by Jian et al. [43], who generated 13 potential high-entropy perovskite candidates. In their work, Ba^2+^ or Sr^2+^ were positioned in the A site, while the B site accommodated five cations in an equimolar ratio. The selection of these cations aimed to achieve a Goldschmidt’s tolerance factor t ranging from 0.95 to 1.05. Tolerance factor is a geometric parameter employed to predict the stability and symmetry of perovskite structures. It is defined as a function of the ionic radii of the A-site cation, $R_{A}$, B-site cation, $R_{B}$, and the oxygen anion, $R_{O}$ [44]:

(2) $$t=\frac{R_{A}+R_{O}}{\sqrt{2}\;(R_{B}+R_{O})},$$

An ideal cubic perovskite typically has a tolerance factor in the range 0.9 ≤t≤ 1.0. When t > 1, hexagonal or tetragonal distortions may occur, while values of t < 0.9 often lead to orthorhombic or rhombohedral structures [43,45]. However, this classical tolerance factor only accurately distinguishes between perovskite and non-perovskite phases for about 74% of known materials [46], making it a useful but imperfect predictive tool. More recently, Bartel et al. [46] using machine learning in combination with physical arguments, proposed a new tolerance factor that improves the prediction of perovskite formation up to 91%.

Despite its limitations, the tolerance factor remains the most widely used parameter for assessing perovskite stability. In systems with mixed A- and B-site cations, such as HEOs, a generalized form of the tolerance factor, $t_{avg}$, is used. It incorporates the weighted average ionic radii of the constituent cations and is expressed as follows [43,47]:

(3) $$t_{avg}=\frac{\left(\sum_{i=1}^{m}x_{A_{i}}R_{A_{i}}\right)/\sum_{i=1}^{m}x_{A_{i}}+R_{O}}{\sqrt{2}\;\left(\left(\sum_{i=1}^{n}x_{B_{i}}R_{B_{i}}\right)/\sum_{i=1}^{n}x_{B_{i}})\right)+R_{O}},$$

where i indexes the different types of cations at each site: i = 1, 2, …, m for A-site cations and i = 1, 2, …, n for B-site cations. The variables $x_{A_{i}}$ and $x_{B_{i}}$ represent the atomic fractions of each corresponding cation, while $R_{A_{i}}$ and $R_{B_{i}}$ are their ionic radii. Although it has been reported that $t_{avg}\sim$1 serves as a significant criterion for the development of a single-phase P-HEO, it is not the only determining factor [48].

Sarkar et al. [48] investigated various P-HEOs, including five trivalent cations on the A and/or B sites. Their successful design resulted in the high-entropy system (GdLaNdSmY)_1_(CoCrFeMnNi)_1_O_3_, confirming the role of entropy in sustaining the phase stability of a single-phase solid solution. In the search for new P-HEOs, Tang et al. [49] and Ma et al. [50] developed a systematic method that examines all possible cation combinations with different valence states to maintain electroneutrality within the perovskite structure. Their findings revealed no discernible link between the emergence of superstructure peaks, indicative of a perovskite with lower symmetry, and the disparity in cation sizes. Instead, Ma et al. [50] found that in non-equimolar compounds, there is no clear relationship between the formation of a single phase and the $t_{avg}$ factor. Conversely, they confirmed a correlation between valence mismatch and the distortion of the superstructure.

### 2.4. High-Entropy Oxides with Pyrochlore, Py-HEO, and Fluorite, F-HEO, Structures

In oxides with an A_2_B_2_O_7_ pyrochlore-type structure, the A sites typically accommodate RE, alkaline, or alkaline–earth cations, while the B sites host transition or post-transition cations. The pyrochlore structure shares similarities with fluorite and bixbyite, as it is both derived from the fluorite-type framework and capable of undergoing structural transformations into a fluorite phase under specific conditions [51,52].

Although pyrochlore, Py, and fluorite, F, structures have the same cation arrangements at the A and B sites, differences in oxygen coordination and the intrinsic presence of oxygen vacancies give rise to intriguing phase transition behaviors. For example, the incorporation of 3+ and 5+ cations often promotes the local formation of the Py-phase, a tendency that diminishes with increasing temperature [53]. Studies by Wright et al. [54] have shown that ordered Py-structures can transform into disordered F-structures. This order–disorder transition is not mainly governed by configurational entropy, but rather by factors such as differences in average A/B ionic radii. In fact, the structural stability of Py-type compounds is intricately linked to the ratio of the average ionic radii of A and B cations, $r_{A}/r_{B}$ [55]. The formation of a Py-structure is favored when $r_{A}/r_{B}$ falls within the range of 1.46 to 1.78. If $r_{A}/r_{B}$ is less than 1.46, a defect fluorite structure tends to be formed; when the value exceeds 1.78, a single cubic phase can no longer be achieved. This criterion has been extensively confirmed for Py-HEOs.

Initial Py-HEO compositions featured five RE cations in the A site and Zr in the B site, such as (LaCeNdSmEu)_2_Zr_2_O_7_ and (LaNdSmEuGd)_2_Zr_2_O_7_ [56,57], and similarly, compositions featuring a single cation at the A site and five different elements at the B site have been reported, such as Nd_2_(TaScSnHfZr)_2_O_7_ [58]. The range of compositionally complex pyrochlores was ultimately broadened by introducing multiple cations into both the A and B sites, covering a range of compositions from high to medium entropy [58]. Teng et al. [12] synthesized 37 distinct equiatomic compositions, of which 30 were classified as Py-HEOs and 7 as F-HEOs, of which 33 exhibited a single-phase structure. It was found that the secondary phases were primarily associated with Ce^4+^ and Nb^5+^. This behavior is attributed to the significantly larger ionic radius of Ce^4+^ compared to other B-site cations, and the distinct oxidation state of Nb^5+^ relative to the typical +4 state. The study highlights that phase stability in these systems is predominantly influenced by the ionic radii and valence states of the constituent cations.

In 2017, Djenadic et al. [59] provided some of the earliest evidence for the formation of F-HEOs, successfully producing equiatomic, single-phase materials that contain up to seven rare-earth elements. The rare-earth cations used included Ce, La, Nd, Pr, Sm, Y, and Gd. Their study revealed that the presence of cerium was essential for achieving a single-phase fluorite structure, regardless of the total number of elements present. Indeed, equiatomic mixtures containing three, four, five, or six of these cations all formed pure fluorite phases, whereas the seven-component compositions exhibited reduced symmetry, consistent with the formation of a single-phase bixbyite structure. Among the elements tested, only Ce and Pr typically form stable fluorite-type oxides under standard conditions.

The first F-HEO incorporating both transition metals and RE elements was described in 2018 [60], involving various combinations of Hf, Zr, Ce, Y, Yb, Ca, and/or Gd. In that work, eight single-phase F-HEOs were successfully synthesized. Also in 2018, Chen et al. [61] synthesized a fully equiatomic (Ce_0.2_Zr_0.2_Hf_0.2_Sn_0.2_Ti_0.2_)O_2_ F-HEO. Building on these findings, Velasco et al. [62] explored 75 non-equiatomic quinary compositions derived from the equiatomic baseline (Ce_0.2_Pr_0.2_La_0.2_Sm_0.2_Y_0.2_)O_2_. Their study showed that, in addition to Ce^4+^, Pr in its mixed +3/+4 oxidation states also promotes single-phase stability, even in the absence of cerium.

## 3. Descriptors

Various descriptors have been formulated to assess the phase stability of high-entropy materials, similar to the well-established Hume-Rothery rules used for binary solid solutions [63]. These descriptors are typically classified into three categories, each providing a distinct perspective on configurational characterization.

### 3.1. Descriptors Based on Entropy and Enthalpy

Thermodynamic parameters, particularly entropy and enthalpy, are naturally suited as descriptors for characterizing structural disorder. These parameters are based on the Gibbs free energy, usually calculated by assessing the mixing enthalpy and entropy changes. Of these, configuration entropy is one of the most commonly utilized descriptors, though it often neglects the potential influence of oxygen vacancies. A simplified expression for this entropy is provided in [64]:

(4) $$∆S_{mix}=-R\left[{\left(\sum_{i=1}^{N}x_{i}\ln x_{i}\right)}_{Cation-site}+{\left(\sum_{j=1}^{L}x_{j}\ln x_{j}\right)}_{Anion-site}\right],$$

In the given expression, $x_{i}$ and $x_{j}$ denote the mole fraction of elements residing in the cation and anion sites, respectively. It is important to highlight that Equation (4) does not consider the configuration entropy arising from interactions between sublattices in oxides. This formulation highlights the flexibility inherent in tailoring HEOs. When compositional complexity involves two distinct cation sublattices, a minimum of five different cation elements is generally required. This multi-sublattice diversity enhances both stability and structural robustness, making it essential for entropy-based materials design. Furthermore, for achieving specific functionalities, compositional complexity across different sublattices is often necessary.

Moving beyond the use of entropy alone, Pitike et al. [21] proposed descriptors based on the statistical mean and standard deviation of local mixing enthalpies. Sarker et al. [15] introduced the entropy-forming ability (EFA) descriptor, which quantifies the energy distribution width of a unit cell randomly sampled with different elemental configurations. A narrower distribution suggests a system that more easily adopts a disordered configuration, thereby favoring the formation of high-entropy phases. The EFA is defined as follows:

(5) $$EFA={\left(\sqrt{\frac{\sum_{i=1}^{N}g_{i}{\left(H_{i}-H_{mix}\right)}^{2}}{\left(\sum_{i=1}^{N}g_{i}\right)-1}}\right)}^{-1}$$

where N is the total number of sampled geometrical configurations, $g_{i}$ represents the degeneracy of each configuration, $H_{i}$ is the enthalpy of configuration i, and $H_{mix}$ is the average mixed-phase enthalpy, given by the following:

(6) $$H_{mix}=\frac{\sum_{i=1}^{N}g_{i}H_{i}}{\sum_{i=1}^{N}g_{i}}$$

Unlike purely entropy-based descriptors, EFA incorporates enthalpic effects to describe the single-phase stability using density functional theory (DFT) calculations. However, its application may be limited due to the high computational cost.

To further refine the thermodynamic description, Curtarolo et al. [65] proposed the Density of Energy Ensemble Descriptor (DEED). They modeled the systematic energy landscape as a thermodynamic density of states spectrum, Ω(E)δ(E), representing a continuous population of configurations. Random distributions were approximated using an ensemble average of ordered representative states, termed partial occupation (POcc), evaluated via DFT. The DEED descriptor balances entropy gain and enthalpy cost, and is defined as follows:

(7) $$DEED=\sqrt{\frac{\sigma_{\Omega }^{-1}\left|H_{f}\right|}{{\langle \Delta H_{f,full}\rangle }_{\Omega }}}$$

where $H_{f}$ is the DFT formation energy of the POcc configurations, $H_{f,full}$ represents the formation enthalpy relative to the convex hull, and $\sigma_{\Omega }$ is the standard deviation of the energy distribution.

### 3.2. Disorder Factor for Different Structures

In addition to thermodynamic descriptors, various disorder factors have been developed to quantify lattice distortion and atomic-level deviations. These factors offer a more structural and geometric perspective on disorder, complementing the energy-based descriptors.

One of the earliest and most widely used metrics is the size disorder parameter, $\delta_{r}$, introduced by Zhang et al. [66], for multicomponent alloys. It captures the deviation in atomic radii among constituent elements and is defined as follows:

(8) $$\delta_{r}=\;\sqrt{\sum_{i=1}^{n}x_{i}{\left(1-\frac{r_{i}}{\bar{r}}\right)}^{2}}$$

where $x_{i}$ and $r_{i}$ are the atomic fraction and atomic radius of element i, respectively, and $\bar{r}$ is the average atomic radius of all constituents. According to Miracle et al. [67], a threshold value of $\delta_{r}$ < 6.5% is generally required for the formation of stable single-phase HEAs. Though originally developed for HEAs, this parameter has since been adapted for HEOs, where it provides insights into phase stabilization based on the size disparity of cations.

In addition to size disorder, the mass disorder factor g has also been used to evaluate compositional disorder in HEOs. These are defined as follows:

(9) $$g=\;\sum_{i=1}^{n}x_{i}{\left(1-\frac{m_{i}}{\bar{m}}\right)}^{2}$$

where $m_{i}$ is the atomic mass of element i, and $\bar{m}$ is the average atomic mass.

When dealing with materials that have multiple sublattices (e.g., A and B cation sites in perovskite or spinel structures), these disorder factors can be evaluated separately for each sublattice and then combined into an overall disorder metric. The composite expressions are as follows:

(10) $$g^{*}=\;\sqrt{g_{A}^{2}+g_{B}^{2}}$$

(11) $${\delta_{r}}^{*}=\sqrt{\delta_{A}^{2}+\delta_{B}^{2}}$$

where $g_{A}$, $g_{B}$, $\delta_{A}$, and $\delta_{B}$ represent the mass and size disorder factors corresponding to the A-site and B-site sublattices, respectively.

These geometric disorder factors offer complementary insight to thermodynamic models by emphasizing structural compatibility, lattice distortion, and the feasibility of forming homogeneous solid solutions in complex oxide systems.

### 3.3. Classical Structure Descriptor for Different Structures

Beyond thermodynamic and disorder-related descriptors, researchers have also employed classical crystallographic parameters to evaluate the structure and phase stability of high-entropy materials. For instance, the tolerance factor (Equation (4)) is commonly used for perovskite structures, while the standard deviation of ionic radii or the radius ratio (e.g., rArB) is typically applied to fluorite and pyrochlore structures.

**Figure 1:**
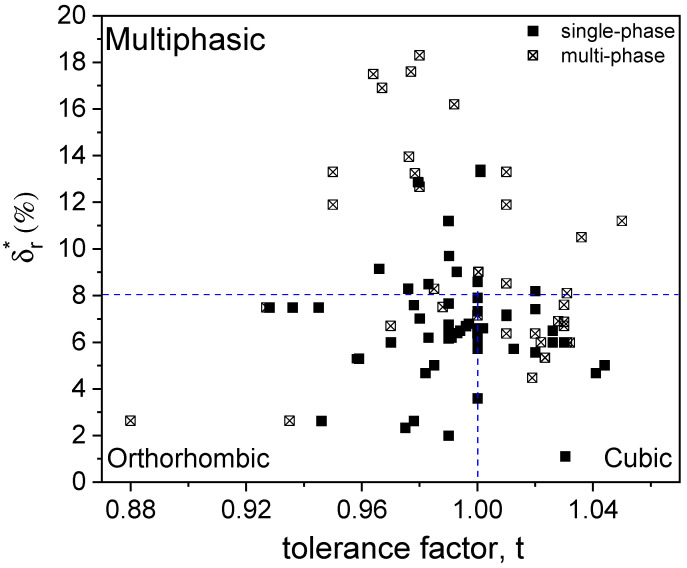
Cation size mismatch effect vs. Goldschmidt’s tolerance factor of perovskite high-entropy oxides. Data compiled from [43,48,68,69,70,71,72,73,74,75,76,77,78,79,80,81]. Filled symbols indicate single-phase formation, while empty ones denote multi-phase systems.

As illustrated in Figure 1, a relationship can be observed between lattice structures of various P-HEOs, the tolerance factor, and cation size disorder. The data, compiled from references listed in the figure caption, show that for single-phase P-HEOs, a tolerance factor close to 1 is crucial in distinguishing orthorhombic from cubic perovskites. Specifically, perovskites tend to form stable phases when 0.92 ≤t≤ 1.04.

On the other hand, when the effective size disorder $\delta_{r}^{*}$ reaches or exceeds 8%, the probability of forming dual-phase ceramics—such as a mixture of cubic and orthorhombic phases or other secondary phases—increases significantly. Notably, this threshold is higher than the commonly accepted limits for forming single-phase HEAs. Yet experimental evidence shows that single-phase P-HEOs with $\delta_{r}^{*}$ as high as 13% can still be achieved. This suggests that cation size mismatch, while important, is not a sufficient condition on its own to guarantee a single-phase solid solution. Moreover, the tolerance factor alone cannot reliably predict whether a near-unity system will form a cubic or tetragonal perovskite phase.

**Figure 2:**
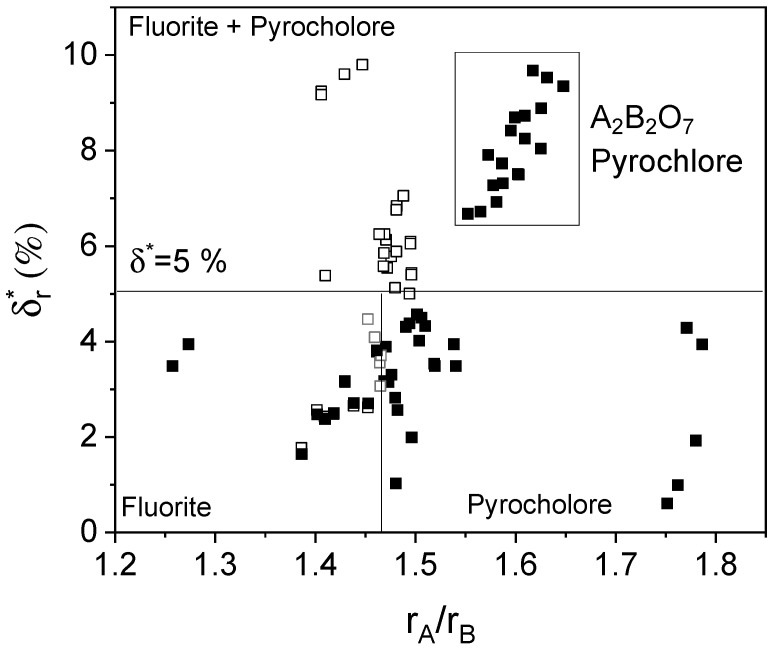
Atomic size effect vs. the average ionic radius ratio of *A* and *B* cations. Data taken from [56,57,58,82,83,84,85,86,87,88,89,90,91,92]. Each symbol corresponds to a different reference. Filled and empty symbols indicate single- and multi-phase formation.

Figure 2 shows the relationship between lattice structures, average ionic radius ratios, and size disorder for A or B cations in Py-HEOs. For single-phase Py-HEOs with disorder on only one sublattice, an average ionic radius ratio close to 1.46 is a critical threshold distinguishing pyrochlore from fluorite structures. When the size disorder reaches or exceeds 5%, the formation of dual-phase systems becomes increasingly likely. In systems containing multiple cations in both sublattices, the range of atomic size disorder consistent with single-phase formation is shown as the marked region in Figure 2.

Despite the utility of these classical descriptors, it is important to note that no universally reliable descriptor currently exists to fully capture the complex interplay between entropy, structure, and stability in HEO systems. Most existing descriptors offer valuable insights only under specific compositional or structural contexts, and many lack a strong physical foundation that generalizes across material families.

## 4. Unified Descriptors

Among the parameters that promote the formation of high-entropy systems, two are particularly important: configurational entropy and the atomic size deviation. In the case of HEAs, which are simple solid solutions with basic crystal structures and monoatomic motifs, the significance of the mixing entropy $∆S_{mix}$ is clear: increasing the number of equivalent atomic configurations raises the entropy, which thermodynamically favors disordered solid solutions over intermetallic compounds or segregated phases, which may have lower entropy but often possess lower free energy.

The relevance of the atomic size deviation $\delta_{r}$ lies in the fact that its square is proportional to the elastic strain energy stored in the system. As the composition diverges from a pure element and the size mismatch among atoms increases, the lattice becomes increasingly distorted, leading to a higher degree of stored strain energy.

**Figure 3:**
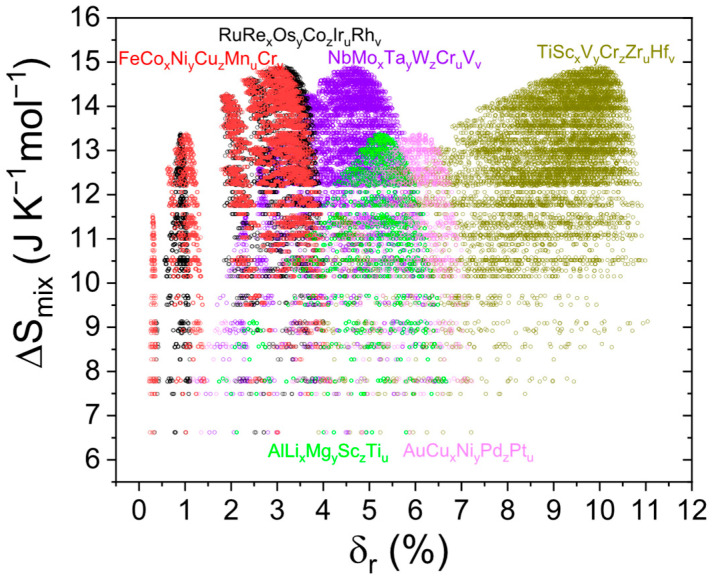
$∆S_{mix}$ as a function of $\delta_{r}$ for different HEA families.

Figure 3 presents the relationship between mixing entropy and atomic size deviation for a series of theoretical HEA compositions, grouped by alloy families. As expected, the maximum entropy corresponds to equimolar compositions. As one moves away from this ideal composition, the entropy decreases. The behavior of $\delta_{r}$, on the other hand, may either increase or decrease depending on the specific elements in the system, as their individual atomic sizes govern how $\delta_{r}$ evolves. The figure highlights different alloy families that crystallize in distinct structures. For HEAs, this analysis is feasible because both $\delta_{r}$ and $∆S_{mix}$ have unambiguous definitions.

In Equation (4), the definition of configuration entropy is intensive but not properly normalized to one mole of the system, as it actually corresponds to at least two moles, depending on the number of cationic sites present in the structure under consideration. For instance, comparing the rock salt and the spinel AB_2_O_4_ structures, consider an oxide with n different cations randomly distributed among the available cation sites. In such a scenario, the entropy contribution for each cation site would be $ln(\frac{1}{n})$, while the oxygen site, with no configuration disorder, does not contribute. Thus, the total configuration entropy becomes $∆S_{mix}=Rln(n)$ and $∆S_{mix}=2Rln(n)$ for the rock salt and the spinel structures, respectively. Therefore, even though the spinel structure has two distinct types of cation sites, the total entropy appears artificially higher than that of the rock salt structure, due to improper normalization. This discrepancy stems from not correctly defining what constitutes one mole of the system, leading to an overestimation of the entropy. The correct normalization should consider one mole of the material, accounting for all atoms, including oxygen. Accordingly, the expression for the configuration entropy should be as follows:

(12) $$\begin{array}{cc} ∆S_{mix}^{N} & =-R[\frac{c_{A}}{c_{A}+c_{B}+c_{o}}(\sum_{h=1}^{M}x_{h}\ln x_{h}{)}_{A-site}\\ & +\frac{c_{B}}{c_{A}+c_{B}+c_{o}}(\sum_{i=1}^{N}x_{i}\ln x_{i}{)}_{B-site}\\ & +\frac{c_{o}}{c_{A}+c_{B}+c_{o}}(\sum_{j=1}^{L}x_{j}\ln x_{j}{)}_{Anion-site}] \end{array}$$

This expression accounts for the fraction of A sites, B sites, and oxygen sites, denoted as $c_{A}$, $c_{B}$, and $c_{o}$, respectively. In the case of complete disorder of the various elements across their respective sites, the configuration entropy becomes, for the rock salt structure, $∆S_{mix}^{N}=\frac{R}{2}ln(n)$, and, for the spinel structure, $∆S_{mix}^{N}=\frac{3R}{7}ln(n)$. This yields a more physically consistent result. In the rock salt structure, as a maximum, 50% of the atomic sites are involved in the disorder, while in the spinel structure, at most 43% of the sites contribute to configuration disorder.

In the case of $∆S_{mix}^{N}$, the expression is normalized and directly reflects compositional disorder. However, for $\delta_{r}^{*}$, as expressed in Equation (8), the lack of normalization tends to overestimate the structural distortions. These distortions are more significant when extended over larger volumes, and thus, the distortion energy should be volume-weighted for greater accuracy. To account for this, a corrected form of $\delta_{r}^{N}$ can be defined as follows:

(13) $$\delta_{r}^{N}=\sqrt{\frac{n_{A}\Omega_{A}}{\Omega_{cell}}{\delta_{r}\left(A\right)}^{2}+\frac{n_{B}\Omega_{B}}{\Omega_{cell}}{\delta_{r}\left(B\right)}^{2}}$$

where $n_{X}$ is the number of X-site atoms (X = A or B) per unit cell, $\Omega_{X}$ is the volume of the coordination polyhedron at site X, and $\Omega_{cell}$ is the total unit cell volume.

**Figure 4:**
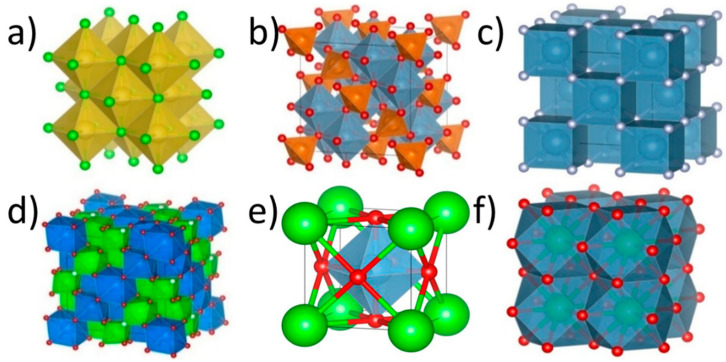
Polyhedral coordination of different crystal structures exhibited by HEOs: (**a**) rock salt, (**b**) spinel, (**c**) fluorite, (**d**) pyrochlore, and (**e**,**f**) perovskite.

For example, in a rock salt structure, each cation is surrounded by an octahedron that occupies one-quarter of the cell’s volume (see Figure 4). With four cations per unit cell, the relative polyhedral volume becomes $\Omega_{X}/\Omega_{cell}$ = 1, meaning the correction has no impact.

In contrast, for a spinel structure, there are 8 tetrahedral A-sites (each occupying 1/192 of the cubic cell) and 16 octahedral B-sites (each occupying 1/32). Applying these values, the corrected expression becomes the following:

(14) $$\delta_{r}^{N}=\sqrt{\frac{8}{192}{\delta_{r}\left(A\right)}^{2}+\frac{16}{32}{\delta_{r}\left(B\right)}^{2}}=\;\sqrt{\frac{1}{24}{\delta_{r}\left(A\right)}^{2}+\frac{1}{2}{\delta_{r}\left(B\right)}^{2}}$$

This reveals that structural distortions introduced at the A-site are less energetically penalized due to their smaller spatial contribution, making it easier to accommodate size mismatches on these sites. Table 1 provides the correction coefficients for various sublattices and structure types.

**Figure 5:**
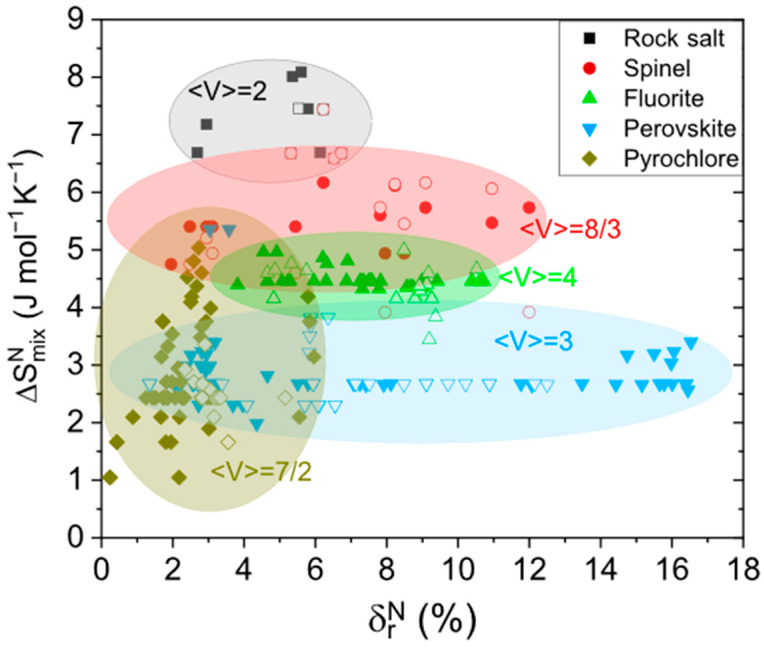
Atomic size effect vs. the average ionic radius ratio of *A* and *B* cations. Experimental composition taken from [12,21,27,30,31,32,34,36,37,43,48,50,68,70,71,72,75,76,78,81,82,86,87,88,89,90,92,93,94,95,96,97,98,99,100,101]. Filled and empty symbols indicate single- and multi-phase formation, respectively. <V> represents the mean cation valence of the indicated structure.

Figure 5 presents the relationship between $∆S_{mix}^{N}$ and $\delta_{r}^{N}$ for different crystal structures, with the goal of differentiating between the formation of single-phase and multi-phase systems. The shaded areas in the figure indicate the typical ranges where each structure tends to exist. The corresponding experimental compositions and the obtained data for each composition can be found in Table S1.

It can be observed that the rock salt structure occupies the existence range with the highest $∆S_{mix}^{N}$, which is attributed to its lower oxygen content. Specifically, it lies within the range of approximately 6.5–8 J mol^−1^ K^−1^. In contrast, the other analyzed structures are generally found within a broader but lower $∆S_{mix}^{N}$ range of 1–6.5 J mol^−1^ K^−1^. Although $∆S_{mix}^{N}$ does not allow for a clear distinction between monophasic and multiphasic formations, it does help to differentiate structural types within defined entropy ranges.

Regarding $\delta_{r}^{N}$, different structures exhibit varying degrees of flexibility. The pyrochlore structure shows the least tolerance to cationic size mismatch; no single-phase compounds have been reported with $\delta_{r}^{N}$ > 2%, and multi-phase formations tend to appear beyond this threshold. In contrast, the perovskite structure demonstrates the highest admittance, with stable single-phase compounds observed even at $\delta_{r}^{N}$ values approaching 16%. Generally, structures with multiple cation sublattices tend to accommodate greater distortions, thereby enabling the formation of monophasic compounds over a wider $\delta_{r}^{N}$ range. Based on the experimental data presented in Figure 5, the following specific observations can be made: (1) As is well known, spinel and rock salt structures often compete with each other. To favor the formation of a rock salt structure, values of $\delta_{r}^{N}$ between 3 and 6 should be considered. The spinel structure demonstrates an even higher tolerance to misfit. (2) Although the perovskite structure displays the highest overall misfit admittance, the formation of a single-phase perovskite is limited to two specific 3 $<\delta_{r}^{N}<$ 4 and 14 $<\delta_{r}^{N}<$ 17 ranges. (3) For the pyrochlore structure, single-phase formation occurs when $\delta_{r}^{N}<6.$ (4). The fluorite structure, which competes with pyrochlore, forms within a broader range, 4 $<\delta_{r}^{N}<$ 11. The two distinct regions where single-phase perovskite structures are observed may be related to the presence of two sublattices within the perovskite structure, as well as the wide range of distortions it can accommodate (tetragonal, cubic, and orthorhombic).

This joint analysis of phase formation based on $∆S_{mix}^{N}$ and $\delta_{r}^{N}$ allows a comparative study across different structure types. Although these parameters are critical for predicting phase stability in HEOs, their individual or combined use does not fully guarantee the formation of a single-phase solid solution or its crystal structure type. Additional factors, particularly the synthesis method and the average oxidation state per cation, can significantly influence phase formation. The mean cation valence, <V>, which is characteristic of each structure, offers valuable insight into the type of structure that might be formed with a given combination of cations. This provides a useful first approximation for predicting structural outcomes.

Despite the limitations of the analysis, the corrected values of $∆S_{mix}^{N}$ and $\delta_{r}^{N}$ provide a useful first-order estimation of the type of structure likely to form from a given cation combination, as well as its potential to be single-phase or multi-phase without the need for complex numerical calculations.

## 5. Conclusions

The emerging field of high-entropy oxides introduces a vast new compositional space, offering the potential for new materials with enhanced properties. Recent active research in high-entropy materials and the development of predictive tools have generated a sufficiently large database to discern certain general trends. This study investigates the primary factors influencing the phase stability of important oxide crystal structures. High-entropy rock salt, spinels, perovskites, fluorites, and pyrochlores demonstrate significant compositional and chemical versatility, with stable frameworks able to incorporate a diverse array of elements. Achieving phase stabilization in these materials involves the careful choice of cations, taking into account the balance of their concentrations, valence states, and ionic sizes.

A unified framework is proposed for analyzing the stability of HEOs, incorporating the normalized configurational entropy per mole of atoms and the relative volume occupied by cations within the mean atomic size deviation. This framework serves as a practical tool for predicting the formation and stability of HEO compounds.

## Figures and Tables

**Table 1:** Volume correction coefficients for $\delta_{r}^{N}$ by structure type.

Structure	$\frac{n_{A}\Omega_{A}}{\Omega_{cell}}$	$\frac{n_{B}\Omega_{B}}{\Omega_{cell}}$
Rock salt	1	-
Fluorite	1/2	-
Spinel (normal)	1/24	1/2
Perovskite	1/6	5/6
Pyrochlore	1/4	$\sqrt{2}$/12

## Data Availability

The dataset is available upon request from the authors.

## Supplementary Materials

The following supporting information can be downloaded at: https://www.mdpi.com/article/10.3390/ma18163862/s1. Table S1. The corresponding experimental compositions and the obtained data for each composition.
