# Supplementary material for: Descriptors for Predicting Single- and Multi-Phase Formation in High-Entropy Oxides: A Unified Framework Approach
Source: Materials (Basel). 2025 Aug 18;18(16):3862. doi: 10.3390/ma18163862 (PMC12387497; doi:10.3390/ma18163862)
Supplement: Supplementary file 1 [file materials-18-03862-s001.zip › materials-3783244-supplementary.pdf]

Descriptors for predicting Single- and Multi-Phase Formation in High Entropy Oxides: A unified framework approach

A.F. Manchón-Gordón[1,*], P. Panadero-Medianero[2] and J.S. Blázquez[2]

[1] *Instituto de Ciencia de Materiales de Sevilla, CSIC-Universidad de Sevilla, C. Américo Vespucio 49, 41092 Sevilla, Spain*
[2] *Dpto. Física de la Materia Condensada, ICMSE-CSIC, Universidad de Sevilla, P.O. Box 1065, 41080 Sevilla, Spain*
* Correspondence: afmanchon@us.es,

Suplementary information

Table S1. The corresponding experimental compositions and the obtained data for each composition.

Composition	Structure	Single-phase	ΔS_{mix}^{N}	δ_r^N	Ref.
(MgCoCuNiZn)O	Rock-salt	Yes	6.69	2.69	[1]
$(MgCoNiCuZn)_{0.95}Li_{0.05}O$		Yes	7.18	2.94	[2]
$(MgCoNiCuZn)_{0.8}(LiGa)_{0.2}O$		Yes	8.01	5.36	
$(Zn_{0.2}Ni_{0.2}Co_{0.2}Mn_{0.2}Fe_{0.2})O$		Yes	6.69	6.13	[3]
$(Zn_{1/6}Ni_{1/6}Co_{1/6}Mn_{1/6}Fe_{1/6}Cu_{1/6})O$		Yes	7.44	5.80	
$(Zn_{1/7}Ni_{1/7}Co_{1/7}Mn_{1/7}Fe_{1/7}Cu_{1/7}Mg_{1/7})O$		Yes	8.09	5.60	
$(Co_{8,3}Cr_{8,6}Fe_{7,8}Mg_{7,2}Mn_{8,1}Ni_{7,7}O_{52,3}$		No	7.44	6.22	[4]
$Co_{11}Fe_{12,1}Mg_{6,2}Mn_{12}Ni_{10,3}O_{48,4}$		No	6.59	6.52	
$Co_{10,6}Cr_{10,3}Fe_{10,4}Mg_{8,6}Ni_{10}O_{50,1}$		No	6.68	5.32	
$Co_{9,7}Cr_{10,8}Mg_{8,6}Mn_{9,9}Ni_{8,9}O_{52,1}$		No	6.68	6.73	
$(Al,Cr,Fe,Mn,Ni)_3O_4$	Spinel	Yes	5.40	5.53	[5]
$(Co,Cr,Al,Mn,Ni)_3O_4$		Yes	5.40	5.46	
$(Co,Cr,Fe,Al,Ni)_3O_4$		Yes	5.40	5.45	
$(Co,Cr,Fe,Mn,Al)_3O_4$		Yes	5.40	5.70	
$(Co,Cr,Fe,Mn,Ni,Al)_3O_4$		Yes	6.11	5.4	
$(Cr,Mn,Fe,Co)_3O_4$		Yes	4.94	2.78	[6]

$(Cr,Mn,Fe,Ni)_3O_4$	Yes	4.94	2.71	
$(Co,Cr,Fe,Mn,Ni)_3O_4$	Yes	5.73	3.13	
$(MgCoMnFeCrNi)_3O_4$	Yes	5.60	2.90	[7]
$(MgCoMnFeCr)_3O_4$	Yes	4.75	2.67	
$(MgCoMnNi)(AlCoMnCrFe)_2O_4$	Yes	5.47	5.30	
$(MgCoMnNiZn)(AlCoMnCrFe)_2O_4$	Yes	5.73	5.20	
$(CoMgMnNiZn)(CoCrFeMnInY)_2O_4$	Yes	6.17	11.21	
$(Co_{8,3}Cr_{8,6}Fe_{7,8}Mg_{7,2}Mn_{8,1}Ni_{7,7}O_{52,3}$	Yes	5.68	2.80	[4]
$Co_{11}Fe_{12,1}Mg_{6,2}Mn_{12}Ni_{10,3}O_{48,4}$	Yes	5.11	2.97	
$Co_{10,6}Cr_{10,3}Fe_{10,4}Mg_{8,6}Ni_{10}O_{50,1}$	Yes	4.91	2.77	
$Co_{9,7}Cr_{10,8}Mg_{8,6}Mn_{9,9}Ni_{8,9}O_{52,1}$	Yes	4.87	2.68	
$Li(Fe_{0,53}MnTi_{0,47})O_4$	Yes	2.47	1.89	[8]
$Li(Fe_{0,6}Mn_{0,85}Ti_{0,55})O_4$	Yes	2.57	1.99	
$Li(Fe_{0,74}Mn_{0,58}Ti_{0,68})O_4$	Yes	2.60	2.12	
$(MgMnFeCrNi)_3O_4$	No	4.75	2.48	[7]
$(MgCoMnNi)(CoMnCrFe)_2O_4$	No	4.94	3.11	
$(MgCoMnNiZn)(CoMnCrFe)_2O_4$	No	5.20	2.95	
$(MgCoFeCrAl)_3O_4$	No	4.60	5.44	
$(MgCoMnFeCrNiIn)_3O_4$	No	6.14	8.23	
$(MgCoMnFeNiIn)_3O_4$	No	5.45	8.48	
$(MgNiCo)(MnFeIn)_2O_4$	No	3.91	7.95	
$(MgCoMnNiZn)(AlCoMnCrFeIn)_2O_4$	No	6.17	9.09	
$(MgCoMnNiZn)(CoMnCrFeIn)_2O_4$	No	5.73	7.82	
$Li(Fe_{0,56}Mn_{0,92}Ti_{0,52})O_4$	No	2.53	1.95	[8]
$Co_{8,3}Cr_{8,6}Fe_{7,8}Mg_{7,2}Mn_{8,1}Ni_{7,7}O_{52,3}$	No	7.44	6.22	[4]
$Co_{11}Fe_{12,1}Mg_{6,2}Mn_{12}Ni_{10,3}O_{48,4}$	No	6.59	6.52	
$Co_{10,6}Cr_{10,3}Fe_{10,4}Mg_{8,6}Ni_{10}O_{50,1}$	No	6.68	5.32	
$Co_{9,7}Cr_{10,8}Mg_{8,6}Mn_{9,9}Ni_{8,9}O_{52,1}$	No	6.68	6.73	

$(GdLaNdSmY)CoO_3$	Perovskite	Yes	2.68	1.35	[9]
$(GdLaNdSmY)FeO_3$		Yes	2.68	1.35	
$Gd(CoCrFeMnNi)O_3$		Yes	2.68	2.73	
$La(CoCrFeMnNi)O_3$		Yes	2.68	2.73	
$Nd(CoCrFeMnNi)O_3$		Yes	2.68	2.73	
$Ba(Mg_{0.17}Zn_{0.17}Ti_{0.21}Nb_{0.22}W_{0.23})O_3$		Yes	2.67	7.91	[10]
$Ba(Mg_{0.15}Yb_{0.17}Ti_{0.21}Nb_{0.23}Ta_{0.24})O_3$		Yes	2.65	12.07	
$Ba(Mg_{0.16}Y_{0.19}Yb_{0.19}Nb_{0.22}W_{0.24})$ O_3		Yes	2.66	15.16	
$Ba(Mg_{0.23}Yb_{0.22}Ti_{0.21}W_{0.17}Mo_{0.17})O_3$		Yes	2.66	14.43	
$Ba(Mg_{0.25}Ti_{0.21}Zr_{0.20}Nb_{0.18}W_{0.16})O_3$		Yes	2.66	7.42	
$Ba(Y_{0.22}Yb_{0.22}Dy_{0.22}W_{0.17}Mo_{0.17})O_3$		Yes	2.66	16.39	
$(Ca_{0.1}La_{0.02}Gd_{0.02}Bi_{0.02}Ba_{0.42}Sr_{0.42})(Co_{0.8}Fe_{0.2})O_3$		Yes	2.82	4.65	[11]
$(Ba_{0.5}Sr_{0.5})(Co_{0.736}Fe_{0.184}Zr_{0.02}Ni_{0.02}Cu_{0.02}Al_{0.02})O_3$		Yes	2.57	16.45	
$(Ca_{0.1}La_{0.02}Gd_{0.02}Bi_{0.02}Ba_{0.42}Sr_{0.42})(Co_{0.736}Fe_{0.184}$Zr0.02$Ni_{0.02}Cu_{0.02}Al_{0.02})O_3$		Yes	3.40	16.53	
$(BaSrCaBiNa)TiO_3$		Yes	2.68	2.57	[12]
$(BaSrCaBiNa)TiO_3$		Yes	2.68	3.07	
$(BaSrCaBiK)TiO_3$		Yes	2.68	2.59	
$(BaCaPbBiNa)TiO_3$		Yes	2.68	2.05	
$(Ba_{1/5}Sr_{1/5}Pb_{1/5}Bi_{1/5}Na_{1/5})TiO_3$		Yes	2.68	2.70	
$(Ba_{1/5}Sr_{1/5}Bi_{1/5}Na_{1/5}K_{1/5})TiO_3$		Yes	2.68	3.27	
$(Ba_{1/5}Ca_{1/5}Bi_{1/5}Na_{1/5}K_{1/5})TiO_3$		Yes	2.68	3.27	
$(Ba_{1/5}Pb_{1/5}Bi_{1/5}Na_{1/5}K_{1/5})TiO_3$		Yes	2.68	2.56	
$(Ba_{1/6}Sr_{1/6}Ca_{1/6}Pb_{1/6}Bi_{1/6}Na_{1/6})TiO_3$		Yes	2.98	2.38	
$(BaSrCaBiNaK)TiO_3$		Yes	2.98	3.04	
$(BaSrCaBi)_{0.2}(NaK)_{0.1}TiO_3$		Yes	2.91	2.92	
$(BaSrCaPbBiNaK)TiO_3$		Yes	3.24	2.81	

$(BaSrCaPbBi)_{1/6}(NaK)_{1/12}TiO_3$	Yes	3.17	2.51	
$Ba(Zn_{0.2}Yb_{0.2}Y_{0.2}W_{0.2}Mo_{0.2})O_3$	Yes	2.68	16.00	[13]
$Ba(Mg_{0.2}Zn_{0.2}Nb_{0.2}Ta_{0.2}W_{0.2})O_3$	Yes	2.68	7.25	
$Ba(Zn_{0.2}Yb_{0.2}Ti_{0.2}Nb_{0.2}W_{0.2})O_3$	Yes	2.68	13.48	
$Ba(Zn_{0.2}Ti_{0.2}Zr_{0.2}Hf_{0.2}W_{0.2})O_3$	Yes	2.68	8.11	
$Ba(Zn_{0.2}Ti_{0.2}Zr_{0.2}Nb_{0.2}Ta_{0.2})O_3$	Yes	2.68	7.07	
$Ba(Yb_{0.2}Y_{0.2}Ga_{0.2}Nb_{0.2}W_{0.2})O_3$	Yes	2.68	16.40	
$Ba(Yb_{0.2}Y_{0.2}Ti_{0.2}Zr_{0.2}W_{0.2})O_3$	Yes	2.68	15.65	
$Ba(Yb_{0.2}Y_{0.2}Ti_{0.2}Nb_{0.2}Ta_{0.2})\ O_3$	Yes	2.68	15.77	
$Ba(Ti_{0.2}Zr_{0.2}Hf_{0.2}Sn_{0.2}Ce_{0.2})O_3$	Yes	2.68	11.77	
$(Sr_{0.25}Ca_{0.25}Ba_{0.25}Nd_{0.25})TiO_3$	Yes	2.68	10.88	[14]
$(Sr_{0.25}Ca_{0.25}Ba_{0.25}Sm_{0.25})TiO_3$	Yes	2.31	3.69	
$(Sr_{0.25}Ca_{0.25}Ba_{0.25}Eu_{0.25})TiO_3$	Yes	2.31	3.96	
$(Gd_{0.2}La_{0.2}Nd_{0.2}Sm_{0.2}Y_{0.2})(Co_{0.2}Cr_{0.2}Fe_{0.2}Mn_{0.2}Ni_{0.2})O_3$	Yes	5.35	3.04	[15]
$(Gd_{0.2}Sr_{0.2}Nd_{0.2}Sm_{0.2}Y_{0.2})(Co_{0.2}Cr_{0.2}Fe_{0.2}Mn_{0.2}Ni_{0.2})O_3$	Yes	5.35	3.58	
$SrCe_{1/7}Sn_{1/7}Zr_{1/7}Ti_{1/7}Y_{1/7}Nb_{1/7}Al_{1/7}O_3$	Yes	3.24	16.06	[16]
$SrCe_{1/6}Sn_{1/6}Zr_{1/6}Ti_{1/12}Y_{1/6}Nb_{1/6}Al_{1/8}O_3$	Yes	3.20	15.49	
$SrCe_{1/12}Sn_{1/6}Zr_{1/6}Ti_{1/6}Y_{1/12}Nb_{1/6}Al_{1/6}O_3$	Yes	3.17	14.74	
$SrCe_{1/12}Sn_{1/12}Zr_{1/5}Ti_{1/5}Y_{4/15}Nb_{1/12}Al_{1/12}O_3$	Yes	3.03	15.97	
$Ba(ZrTiSnHfTa)O_3$	Yes	2.68	5.94	[17]
$(Na_{0.2}Bi_{0.2}Ba_{0.2}Sr_{0.2}Ca_{0.2})TiO_3$	Yes	2.68	2.57	[18]
$(Na_{0.24}Bi_{0.19}Ba_{0.19}Sr_{0.119}Ca_{0.19})(Ti_{0.95}Nb_{0.05})O_3$	Yes	3.00	2.95	
$(Na_{0.28}Bi_{0.18}Ba_{0.18}Sr_{0.18}Ca_{0.18})(Ti_{0.90}Nb_{0.10})O_3$	Yes	3.18	2.94	
$(Na_{0.32}Bi_{0.17}Ba_{0.17}Sr_{0.17}Ca_{0.17})(Ti_{0.85}Nb_{0.15})O_3$	Yes	3.40	3.08	
$(Na_{0.36}Bi_{0.16}Ba_{0.16}Sr_{0.16}Ca_{0.16})(Ti_{0.80}Nb_{0.20})O_3$	Yes	3.40	3.18	
$(La_{0.2}Nd_{0.2}Sm_{0.2}Y_{0.2}Gd_{0.2})MnO_3$	Yes	2.68	1.35	[19]
$(La_{0.2}Nd_{0.2}Pr_{0.2}Sr_{0.2}Ba_{0.2})MnO_3$	Yes	2.68	3.28	

$(La_{0.2}Nd_{0.2}Sm_{0.2}Ca_{0.2}Sr_{0.2})MnO_3$		Yes	2.68	2.16	
$La_{0.2}Pr_{0.2}Nd_{0.2}Sm_{0.2}Sr_{0.2}MnO_3$		Yes	2.68	2.22	[20]
$(Bi_{0.2}Na_{0.2}Ba_{0.2}Sr_{0.2}Ca_{0.2})TiO_3$		Yes	2.68	2.57	[21]
$(Bi_{0.2}Li_{0.2}Ba_{0.2}Sr_{0.2}Pb_{0.2})TiO_3$		Yes	2.68	3.25	
$(Bi_{0.2}Na_{0.2}Ba_{0.2}Sr_{0.2}Pb_{0.2})TiO_3$		Yes	2.68	2.10	
$(Bi_{0.2}K_{0.2}Ba_{0.2}Sr_{0.2}Pb_{0.2})TiO_3$		Yes	2.68	2.22	
$(Bi_{0.2}Ag_{0.2}Ba_{0.2}Sr_{0.2}Pb_{0.2})TiO_3$		Yes	2.68	2.85	
$La(CoFeNiCrAl)O_3$		Yes	2.68	5.51	[22]
$Pr(CrMnFeCoNi)O_3$		Yes	2.68	5.78	[23]
$Ba(Zr_{0.25}Sn_{0.25}Hf_{0.25}Nb_{0.25})O_3$		No	2.31	4.08	[17]
$Ba(Zr_{0.25}Ti_{0.25}Sn_{0.25}Ta_{0.25})O_3$		No	2.31	6.10	
$Ba(Zr_{0.25}Ti_{0.25}Hf_{0.25}Ta_{0.25})O_3$		No	2.31	6.55	
$Ba(Ti_{0.25}Sn_{0.25}Hf_{0.25}Ta_{0.25})O_3$		No	2.31	5.70	
$Ba(Zr_{0.25}Sn_{0.25}Hf_{0.25}Ta_{0.25})O_3$		No	2.31	4.08	
$Ba(Zr_{0.25}Ti_{0.25}Sn_{0.25}Hf_{0.25})O_3$		No	2.31	6.07	
$(La_{0.2}Pt_{0.2}Ca_{0.2}Sr_{0.2}Ba_{0.2})MnO_3$		No	2.68	3.39	[19]
$(La_{0.2}Nd_{0.2}Ca_{0.2}Sr_{0.2}Ba_{0.2})MnO_3$		No	2.68	3.39	
$Pr_{0.9}Sr_{0.1}(CrMnFeCoNi)O_3$		No	3.22	5.82	[23]
$Pr_{0.8}Sr_{0.2}(CrMnFeCoNi)O_3$		No	3.51	5.84	
$Pr_{0.7}Sr_{0.3}(CrMnFeCoNi)O_3$		No	3.69	5.86	
$Pr_{0.6}Sr_{0.4}(CrMnFeCoNi)O_3$		No	3.80	5.87	
$Pr_{0.5}Sr_{0.5}(CrMnFeCoNi)O_3$		No	3.83	5.87	
$CeZrTiSnHfO_2$	Fluorite	Yes	4.46	6.30	[24]
$HfZrCeYYbO_2$		Yes	4.46	5.96	
$ZrCeHfYAlO_2$		Yes	4.46	9.42	
$(HfZrCe)_{0.25}(YSi)_{0.125}O_2$		Yes	4.32	7.32	
$(HfZrCe)_{0.25}$ $(YCa_{0.125}O_2$		Yes	4.32	7.78	

$CeZrTiSnCaO_2$		Yes	4.46	10.62	
$CeZrTiCaHfO_2$		Yes	4.46	10.39	
$CeZrCaSnHfO_2$		Yes	4.46	9.08	
$CeCaTiSnHfO_2$		Yes	4.46	10.71	
$CeLaNdPrSmYO_2$		Yes	4.97	4.91	
$CeGdLaNdPrSmYO_2$		Yes	4.97	4.54	
$CeLaPrSmYO_2$		Yes	4.46	5.06	
$ZrLaSmPrYO_2$		Yes	4.46	7.58	
$CeZrYLaSmO_2$		Yes	4.46	7.50	
$(Ce_{0.2}Zr_{0.2}Er_{0.2}Pr_{0.2}Y_{0.2})O_2$		Yes	4.46	4.65	
$(Pr_{0.2}Zr_{0.2}Hf_{0.2}Y_{0.2}La_{0.2})O_2$		Yes	4.46	9.00	
$(Ce_{0.2}Hf_{0.2}Zr_{0.2}Y_{0.2}Gd_{0.2})O_2$		Yes	4.46	6.88	
$Ce_{7.9}Gd_9Nd_{8.6}Pr_{4.9}Sm_{9.5}O_{60}$		Yes	4.40	3.82	[25]
$Ce_8Gd6.1Nd_{7.4}Pr_{5.5}Sm_{7.5}Mo_{3.2}O_{62}$		Yes	4.86	6.21	
$Ce_{8.9}Gd_{7.5}La_{7.5}Nd_{7.8}Pr_{7.2}O_{61}$		Yes	4.45	5.22	
$Ce_{8.3}Gd_{7.4}La_{7.5}Nd_{8.1}Pr_{6.5}Mo_{1.8}O_{60}$		Yes	4.76	6.32	
$Ce_{7.3}Gd_{7.2}La_7Nd_{7.3}Pr_{6.5}Mo_{2.8}O_{62}$		Yes	4.81	6.90	
$Hf_{0.1}Zr_{0.225}Ce_{0.225}Y_{0.225}Yb_{0.225}O_2$		Yes	4.36	8.57	[26]
$Hf_{0.225}Zr_{0.1}Ce_{0.225}Y_{0.225}Yb_{0.225}O_2$		Yes	4.36	8.66	
$Hf_{0.225}Zr_{0.225}Ce_{0.1}Y_{0.225}Yb_{0.225}O_2$		Yes	4.36	9.01	
$Hf_{0.225}Zr_{0.225}Ce_{0.225}Y_{0.1}Yb_{0.225}O_2$		Yes	4.36	8.74	
$Hf_{0.225}Zr_{0.225}Ce_{0.225}Y_{0.225}Yb_{0.1}O_2$		Yes	4.36	8.95	
$CeZrErPrYO_2$		Yes	4.46	4.65	[27]
$CeZrYPrGdO_2$		Yes	4.46	5.30	
$CeZrYbLaErO_2$		Yes	4.46	7.27	
$CeZrYLaErO_2$		Yes	4.46	7.25	
$CeZrYbLaSmO_2$		Yes	4.46	7.59	
$CeZrYLaGdO_2$		Yes	4.46	7.35	

CeZrYLaSmO $_2$		Yes	4.46	7.50	
CeZrLaSmGdO $_2$		Yes	4.46	7.53	
CeZrNdLaSmO $_2$		Yes	4.46	7.83	
$Ce_{9.5}Gd_{8.8}Nd_9Pr_5Sm_{9.5}Mo_1O_{57}$		No	4.60	4.66	[25]
$Ce_{8.5}Gd_{8.1}Nd_{7.8}Pr_{4.8}Sm_{8.5}Mo_{1.2}O_{61}$		No	4.65	4.87	
$Ce_{7.7}Gd_{7.6}Nd_{7.6}Pr_{6.4}Sm_{8.4}Mo_{1.8}O_{60}$		No	4.76	5.33	
$Ce_{8.3}Gd_{7.4}La_{7.3}Nd_{7.8}Pr_{7.2}Mo_{0.8}O_{61}$		No	4.64	5.75	
$Ce_8Gd_{7.8}La_{7.9}Nd_{8.2}Pr_{4.9}Mo_{1.2}O_{62}$		No	4.67	10.52	
(LaNdEuErYb)ZrO $_2$		No	4.15	4.83	[28]
(LaNdDyErYb)ZrO $_2$		No	4.15	8.82	
$(La_{0.2}Nd_{0.2}Y_{0.2}Er_{0.2}Yb_{0.2})ZrO_2$		No	4.15	8.78	[29]
$(La_{0.2}Nd_{0.2}Sm_{0.2}Gd_{0.2}Yb_{0.2})ZrO_2$		No	4.15	9.26	
$(La_{1/5}Nd_{1/5}Sm_{1/5}Eu_{1/5}Gd_{1/5})_2(Zr_{9/10}Ce_{1/10})_2O_7$		No	4.60	9.17	
$(La_{1/5}Nd_{1/5}Sm_{1/5}Eu_{1/5}Gd_{1/5})_2(Zr_{7/10}Ce_{3/10})_2O_7$		No	5.00	8.48	
$(La_{1/3}Nd_{1/3}Gd_{1/3})_2(Zr_{7/10}Ce_{3/10})_2O_7$		No	4.29	8.94	
$(La_{1/3}Gd_{1/3}Yb_{1/3})_2Zr_2O_7$		No	3.44	9.20	
$(La_{1/4}Nd_{1/4}Gd_{1/4}Yb_{1/4})_2Zr_2O_7$		No	3.84	9.37	
$(La_{1/5}Nd_{1/5}Sm_{1/5}Gd_{1/5}Yb_{1/5})_2Zr_2O_7$		No	4.15	9.26	
$(La_{1/6}Nd_{1/6}Sm_{1/6}Eu_{1/6}Gd_{1/6}Yb_{1/6})_2Zr_2O_7$		No	4.40	9.12	
$(La_{3/20}Nd_{3/20}Sm_{3/20}Eu_{3/20}Gd_{3/20}Yb_{1/4})_2Zr_2O_7$		No	4.37	8.92	
$(La_{1/5}Dy_{1/5}Y_{1/5}Yb_{1/5}Sc_{1/5})_2Zr_2O_7$		No	4.15	8.27	
$(Sm_{1/3}Eu_{1/3}Gd_{1/3})_2(Ti_{1/4}Sn_{1/4}Hf_{1/4}Zr_{1/4})_2O_7$	Pyrochlore	Yes	3.76	1.72	[30]
$(Sm_{1/4}Eu_{1/4}Gd_{1/4}Yb_{1/4})_2(Ti_{1/4}Sn_{1/4}Hf_{1/4}Zr_{1/4})_2O_7$		Yes	4.19	2.52	
$(Sm_{1/3}Eu_{1/3}Gd_{1/3})_2(Ti_{1/2}Sn_{1/6}Hf_{1/6}Zr_{1/6})_2O_7$		Yes	3.53	1.98	
$(Sm_{1/4}Eu_{1/4}Gd_{1/4}Yb_{1/4})_2(Ti_{1/2}Hf_{1/4}Zr_{1/4})_2O_7$		Yes	3.67	2.81	
$(Sm_{3/4}Yb_{1/4})_2(Ti_{1/2}Zr_{1/2})_2O_7$		Yes	1.90	3.00	
$Nd_2(Ta_{0.2}Sc_{0.2}Sn_{0.2}Hf_{0.2}Zr_{0.2})_2O_7$		Yes	2.43	1.25	[31]

$Nd_2(Ti_{0.2}Nb_{0.2}Sn_{0.2}Hf_{0.2}Zr_{0.2})_2O_7$	Yes	2.43	1.52	
$(Sm_{0.2}Eu_{0.2}Tb_{0.2}Dy_{0.2}Lu_{0.2})_2Zr_2O_7$	Yes	2.43	1.84	[32]
$La_2(Zr_{0.2}Ce_{0.2}Hf_{0.2}Sn_{0.2}Ti_{0.2})_2O_7$	Yes	2.43	3.06	[33]
$(LaNdSmEuDy)_2Zr_2O_7$	Yes	2.43	2.07	[34]
$(LaNdSmEuYb)_2Zr_2O_7$	Yes	2.43	2.80	
$(La_{0.2}Nd_{0.2}Sm_{0.2}Eu_{0.2}Gd_{0.2})_2Zr_2O_7$	Yes	2.43	1.73	[35]
$(Y_{0.2}Nd_{0.2}Sm_{0.2}Eu_{0.2}Gd_{0.2})_2Zr_2O_7$	Yes	2.43	1.43	
$(La_{0.2}Y_{0.2}Sm_{0.2}Eu_{0.2}Gd_{0.2})_2Zr_2O_7$	Yes	2.43	2.25	
$(La_{0.2}Nd_{0.2}Y_{0.2}Eu_{0.2}Gd_{0.2})_2Zr_2O_7$	Yes	2.43	2.32	
$(La_{0.2}Nd_{0.2}Sm_{0.2}Y_{0.2}Gd_{0.2})_2Zr_2O_7$	Yes	2.43	2.29	
$(La_{0.2}Nd_{0.2}Sm_{0.2}Eu_{0.2}Y_{0.2})_2Zr_2O_7$	Yes	2.43	2.24	
$Dy_2(Ti_{0.2}Zr_{0.2}Hf_{0.2}Ge_{0.2}Sn_{0.2})_2O_7$	Yes	2.43	2.97	[36]
$(Sm_{0.2}Eu_{0.2}Tb_{0.2}Dy_{0.2}Lu_{0.2})_2Zr_2O_7$	Yes	2.43	1.83	[37]
$(La_{0.2}Nd_{0.2}Sm_{0.2}Gd_{0.2}Yb_{0.2})_2Zr_2O_7$	Yes	2.43	2.82	[38]
$(La_{0.2}Nd_{0.2}Sm_{0.2}Eu_{0.2}Gd_{0.2})_2Zr_2O_7$	Yes	2.43	1.73	[39]
$(La_{1/5}Nd_{1/5}Sm_{1/5}Eu_{1/5}Gd_{1/5})_2Zr_2O_7$	Yes	2.43	1.73	[29]
$(La_{1/3}Nd_{1/3}Gd_{1/3})_2Zr_2O_7$	Yes	1.66	1.95	
$(La_{1/5}Nd_{1/5}Sm_{1/5}Eu_{1/5}Gd_{1/5})_2Hf_2O_7$	Yes	2.43	1.74	
$(Gd_{1/2}Eu_{1/2})_2Zr_2O_7$	Yes	1.05	0.24	[40]
$(Gd_{1/3}Eu_{1/3}Sm_{1/3})_2Zr_2O_7$	Yes	1.66	0.43	
$(Gd_{1/4}Eu_{1/4}Sm_{1/4}Nd_{1/4})_2Zr_2O_7$	Yes	2.09	0.88	
$(Gd_{1/5}Eu_{1/5}Sm_{1/5}Nd_{1/5}La_{1/5})_2Zr_2O_7$	Yes	2.43	1.73	
$(Gd_{1/6}Eu_{1/6}Sm_{1/6}Nd_{1/6}La_{1/6}Dy_{1/6})_2Zr_2O_7$	Yes	2.71	1.97	
$(Gd_{1/7}Eu_{1/7}Sm_{1/7}Nd_{1/7}La_{1/7}Dy_{1/7}Ho_{1/7})_2Zr_2O_7$	Yes	2.94	2.15	
$Gd_2(Ti_{1/2}Zr_{1/2})_2O_7$	Yes	1.04	2.17	[41]
$Gd_2(Ti_{1/3}Zr_{1/3}Sn_{1/3})_2O_7$	Yes	1.66	1.81	
$Gd_2(Ti_{1/4}Zr_{1/4}Sn_{1/4}Hf_{1/4})_2O_7$	Yes	2.09	1.66	
$(Eu_{1/2}Gd_{1/2})_2Ti_2O_7$	Yes	1.04	0.24	

$(Eu_{1/2}Gd_{1/2})_2(Ti_{1/2}Zr_{1/2})_2O_7$	Yes	2.09	2.18	
$(Eu_{1/2}Gd_{1/2})_2(Ti_{1/3}Zr_{1/3}Sn_{1/3})_2O_7$	Yes	2.70	1.82	
$(Eu_{1/2}Gd_{1/2})_2(Ti_{1/4}Zr_{1/4}Sn_{1/4}Hf_{1/4})_2O_7$	Yes	3.14	1.68	
$(Sm_{1/3}Eu_{1/3}Gd_{1/3})_2Ti_2O_7$	Yes	1.66	0.43	
$(Sm_{1/3}Eu_{1/3}Gd_{1/3})_2(Ti_{1/2}Zr_{1/2})_2O_7$	Yes	2.71	2.22	
$(Sm_{1/3}Eu_{1/3}Gd_{1/3})_2(Ti_{1/3}Zr_{1/3}Sn_{1/3})_2O_7$	Yes	3.32	1.86	
$(Sm_{1/3}Eu_{1/3}Gd_{1/3})_2(Ti_{1/4}Zr_{1/4}Sn_{1/4}Hf_{1/4})_2O_7$	Yes	3.76	1.72	
$(Nb_{1/4}Sm_{1/4}Eu_{1/4}Gd_{1/4})_2Ti_2O_7$	Yes	2.09	5.55	
$(Nb_{1/4}Sm_{1/4}Eu_{1/4}Gd_{1/4})_2(Ti_{1/2}Zr_{1/2})_2O_7$	Yes	3.14	5.96	
$(Nb_{1/4}Sm_{1/4}Eu_{1/4}Gd_{1/4})_2(Ti_{1/3}Zr_{1/3}Sn_{1/3})_2O_7$	Yes	3.76	5.83	
$(Nb_{1/4}Sm_{1/4}Eu_{1/4}Gd_{1/4})_2(Ti_{1/4}Zr_{1/4}Sn_{1/4}Hf_{1/4})_2O_7$	Yes	4.19	5.79	
$(La_{1/5}Nd_{1/5}Sm_{1/5}Eu_{1/5}Gd_{1/5})_2(Ti_{1/2}Zr_{1/2})_2O_7$	Yes	3.48	2.78	
$(La_{1/5}Nd_{1/5}Sm_{1/5}Eu_{1/5}Gd_{1/5})_2(Ti_{1/3}Zr_{1/3}Sn_{1/3})_2O_7$	Yes	4.09	2.55	
$(La_{1/5}Nd_{1/5}Sm_{1/5}Eu_{1/5}Gd_{1/5})_2(Ti_{1/4}Zr_{1/4}Sn_{1/4}Hf_{1/4})_2O_7$	Yes	4.53	2.41	
$(La_{1/6}Nd_{1/6}Sm_{1/6}Eu_{1/6}Gd_{1/6}Dy_{1/6})_2(Ti_{1/2}Zr_{1/2})_2O_7$	Yes	3.76	2.94	
$(La_{1/6}Nd_{1/6}Sm_{1/6}Eu_{1/6}Gd_{1/6}Dy_{1/6})_2(Ti_{1/3}Zr_{1/3}Sn_{1/3})_2O_7$	Yes	4.37	2.68	
$(La_{1/6}Nd_{1/6}Sm_{1/6}Eu_{1/6}Gd_{1/6}Dy_{1/6})_2(Ti_{1/4}Zr_{1/4}Sn_{1/4}Hf_{1/4})_2O_7$	Yes	4.80	2.58	
$(La_{1/7}Nd_{1/7}Sm_{1/7}Eu_{1/7}Gd_{1/7}Dy_{1/7}Ho_{1/7})_2Ti_2O_7$	Yes	2.94	2.16	
$(La_{1/7}Nd_{1/7}Sm_{1/7}Eu_{1/7}Gd_{1/7}Dy_{1/7}Ho_{1/7})_2(Ti_{1/2}Zr_{1/2})_2O_7$	Yes	3.99	3.06	
$(La_{1/7}Nd_{1/7}Sm_{1/7}Eu_{1/7}Gd_{1/7}Dy_{1/7}Ho_{1/7})_2(Ti_{1/3}Zr_{1/3}Sn_{1/3})_2O_7$	Yes	4.60	2.81	
$(Gd_{1/7}Eu_{1/7}Sm_{1/7}Nd_{1/7}La_{1/7}Dy_{1/7}Ho_{1/7})_2(Ti_{1/4}Zr_{1/4}Sn_{1/4}Hf_{1/4})_2O_7$	Yes	5.04	2.72	
$(Nd_{1/5}Sm_{1/5}Gd_{1/5}Ho_{1/5}Er_{1/5})_2Zr_2O_7$	Yes	2.43	1.86	
$(LaNdEuErYb)_2Zr_2O_7$	No	2.43	3.17	[34]
$(LaNdDyErYb)_2Zr_2O_7$	No	2.43	3.27	
$(La_{0.2}Nd_{0.2}Y_{0.2}Er_{0.2}Yb_{0.2})_2Zr_2O_7$	No	2.43	3.34	[35]
$(La_{0.2}Nd_{0.2}Sm_{0.2}Gd_{0.2}Yb_{0.2})_2Zr_2O_7$	No	2.43	2.82	

$(La_{1/5}Nd_{1/5}Sm_{1/5}Eu_{1/5}Gd_{1/5})_2(Zr_{9/10}Ce_{1/10})_2O_7$	No	2.92	2.34	[29]
$(La_{1/5}Nd_{1/5}Sm_{1/5}Eu_{1/5}Gd_{1/5})_2(Zr_{7/10}Ce_{3/10})_2O_7$	No	3.36	2.90	
$(La_{1/3}Nd_{1/3}Gd_{1/3})_2(Zr_{7/10}Ce_{3/10})_2O_7$	No	2.58	3.03	
$(La_{1/3}Gd_{1/3}Yb_{1/3})_2Zr_2O_7$	No	1.66	3.55	
$(La_{1/4}Nd_{1/4}Gd_{1/4}Yb_{1/4})_2Zr_2O_7$	No	2.09	3.16	
$(La_{1/5}Nd_{1/5}Sm_{1/5}Gd_{1/5}Yb_{1/5})_2Zr_2O_7$	No	2.43	2.82	
$(La_{1/6}Nd_{1/6}Sm_{1/6}Eu_{1/6}Gd_{1/6}Yb_{1/6})_2Zr_2O_7$	No	2.71	2.59	
$(La_{3/20}Nd_{3/20}Sm_{3/20}Eu_{3/20}Gd_{3/20}Yb_{1/4})_2Zr_2O_7$	No	2.67	2.83	
$(La_{1/5}Dy_{1/5}Y_{1/5}Yb_{1/5}Sc_{1/5})_2Zr_2O_7$	No	2.43	5.16	

References

[1] K.C. Pitike, S. Kc, M. Eisenbach, C.A. Bridges, V.R. Cooper, Predicting the Phase Stability of Multicomponent High-Entropy Compounds, Chemistry of Materials 32(17) (2020) 7507-7515.

[2] D. Bérardan, S. Franger, D. Dragoe, A.K. Meena, N. Dragoe, Colossal dielectric constant in high entropy oxides, physica status solidi (RRL) – Rapid Research Letters 10(4) (2016) 328-333.

[3] L. Lin, K. Wang, R. Azmi, J. Wang, A. Sarkar, M. Botros, S. Najib, Y. Cui, D. Stenzel, P. Anitha Sukkurji, Q. Wang, H. Hahn, S. Schweidler, B. Breitung, Mechanochemical synthesis: route to novel rock-salt-structured high-entropy oxides and oxyfluorides, Journal of Materials Science 55(36) (2020) 16879-16889.

[4] M. Stygar, J. Dąbrowa, M. Moździerz, M. Zajusz, W. Skubida, K. Mroczka, K. Berent, K. Świerczek, M. Danielewski, Formation and properties of high entropy oxides in Co-Cr-Fe-Mg-Mn-Ni-O system: Novel (Cr,Fe,Mg,Mn,Ni)3O4 and (Co,Cr,Fe,Mg,Mn)3O4 high entropy spinels, Journal of the European Ceramic Society 40(4) (2020) 1644-1650.

[5] H. Zhu, H. Xie, Y. Zhao, S. Dai, M. Li, X. Wang, Structure and magnetic properties of a class of spinel high-entropy oxides, Journal of Magnetism and Magnetic Materials 535 (2021) 168063.

[6] J. Cieslak, M. Reissner, K. Berent, J. Dabrowa, M. Stygar, M. Mozdzierz, M. Zajusz, Magnetic properties and ionic distribution in high entropy spinels studied by Mössbauer and ab initio methods, Acta Materialia 206 (2021) 116600.

[7] M. Coduri, M. Fracchia, M. Guerrini, C. Dejoie, P. Ghigna, U.A. Tamburini, Novel In-based high entropy spinel oxides with tunable lattice parameter, Journal of the European Ceramic Society 43(7) (2023) 2728-2739.

[8] D. Callegari, M. Coduri, M. Fracchia, P. Ghigna, L. Braglia, U. Anselmi Tamburini, E. Quartarone, Lithium intercalation mechanisms and critical role of multi-doping in LiFexMn2−x−yTiyO4 as high-capacity cathode material for lithium-ion batteries, Journal of Materials Chemistry C 10(23) (2022) 8994-9008.

[9] A. Sarkar, R. Djenadic, D. Wang, C. Hein, R. Kautenburger, O. Clemens, H. Hahn, Rare earth and transition metal based entropy stabilised perovskite type oxides, Journal of the European Ceramic Society 38(5) (2018) 2318-2327.

[10] J. Ma, K. Chen, C. Li, X. Zhang, L. An, High-entropy stoichiometric perovskite oxides based on valence combinations, Ceramics International 47(17) (2021) 24348-24352.

[11] J. Wang, Z. Cao, X. Zhu, W. Yang, Improving intermediate-temperature stability of BSCF by constructing high entropy perovskites, Journal of Membrane Science Letters 2(2) (2022) 100026.

[12] J. Zhang, S. Liu, Z. Tian, Y. Zhang, Z. Shi, The Formation and Phase Stability of A-Site High-Entropy Perovskite Oxides, Materials 16(6) (2023) 2214.

[13] L. Tang, Z. Li, K. Chen, C. Li, X. Zhang, L. An, High-entropy oxides based on valence combinations: design and practice, Journal of the American Ceramic Society 104(5) (2021) 1953-1958.
[14] P. Zhang, Z. Lou, L. Gong, J. Xu, Q. Chen, M.J. Reece, H. Yan, Z. Dashevsky, F. Gao, High-entropy MTiO3 perovskite oxides with glass-like thermal conductivity for thermoelectric applications, Journal of Alloys and Compounds 937 (2023) 168366.
[15] S. Schweidler, Y. Tang, L. Lin, G. Karkera, A. Alsawaf, L. Bernadet, B. Breitung, H. Hahn, M. Fichtner, A. Tarancón, M. Botros, Synthesis of perovskite-type high-entropy oxides as potential candidates for oxygen evolution, Frontiers in Energy Research Volume 10 - 2022 (2022).
[16] R. Shishkin, V. Zhuravlev, Thermodynamic analysis and phase characterization of high-entropy oxide Sr(Ce0.05Sn0.08Zr0.2Ti0.16Y0.3Nb0.11Al0.1)O2.9: Insights into stability, microstructure, and mechanical properties, Ceramics International 50(9, Part A) (2024) 15348-15355.
[17] S. Zhou, Y. Pu, Q. Zhang, R. Shi, X. Guo, W. Wang, J. Ji, T. Wei, T. Ouyang, Microstructure and dielectric properties of high entropy Ba(Zr0.2Ti0.2Sn0.2Hf0.2Me0.2)O3 perovskite oxides, Ceramics International 46(6) (2020) 7430-7437.
[18] Y. Ning, Y. Pu, C. Wu, S. Zhou, L. Zhang, J. Zhang, X. Zhang, Y. Shang, Enhanced capacitive energy storage and dielectric temperature stability of A-site disordered high-entropy perovskite oxides, Journal of Materials Science & Technology 145 (2023) 66-73.
[19] Y. Shi, N. Ni, Q. Ding, X. Zhao, Tailoring high-temperature stability and electrical conductivity of high entropy lanthanum manganite for solid oxide fuel cell cathodes, Journal of Materials Chemistry A 10(5) (2022) 2256-2270.
[20] Y. Yang, H. Bao, H. Ni, X. Ou, S. Wang, B. Lin, P. Feng, Y. Ling, A novel facile strategy to suppress Sr segregation for high-entropy stabilized La0·8Sr0·2MnO3-δ cathode, Journal of Power Sources 482 (2021) 228959.
[21] Z. Liu, S. Xu, T. Li, B. Xie, K. Guo, J. Lu, Microstructure and ferroelectric properties of high-entropy perovskite oxides with A-site disorder, Ceramics International 47(23) (2021) 33039-33046.
[22] Z. Xu, Z. Du, R. Zhang, F. Zeng, Z. Meng, X. Hu, H. Tian, Regulating the lattice strain field by high-entropy strategy to realize the conformal growth of perovskites for efficient oxygen evolution, Applied Catalysis B: Environment and Energy 344 (2024) 123668.
[23] Z. Zhao, L. Rehder, F. Steinbach, A. Feldhoff, High-Entropy Perovskites Pr1−xSrx(Cr,Mn,Fe,Co,Ni)O3−δ (x = 0–0.5): Synthesis and Oxygen Permeation Properties, Membranes 12(11) (2022) 1123.
[24] G. Chen, C. Li, H. Jia, H. Li, S. Li, B. Gong, L. An, K. Chen, Formation and properties of Ca2+ substituted (Ce0.2Zr0.2Ti0.2Sn0.2Hf0.2)O2 high-entropy ceramics, Journal of the European Ceramic Society 43(6) (2023) 2586-2592.
[25] J. Dąbrowa, M. Szymczak, M. Zajusz, A. Mikuła, M. Moździerz, K. Berent, M. Wytrwal-Sarna, A. Bernasik, M. Stygar, K. Świerczek, Stabilizing fluorite structure in ceria-based high-entropy oxides: Influence of Mo addition on crystal structure and transport properties, Journal of the European Ceramic Society 40(15) (2020) 5870-5881.
[26] J. Sun, L. Guo, Y. Zhang, Y. Wang, K. Fan, Y. Tang, Superior phase stability of high entropy oxide ceramic in a wide temperature range, Journal of the European Ceramic Society 42(12) (2022) 5053-5064.
[27] L. Spiridigliozzi, C. Ferone, R. Cioffi, G. Dell'Agli, A simple and effective predictor to design novel fluorite-structured High Entropy Oxides (HEOs), Acta Materialia 202 (2021) 181-189.
[28] J. Zhu, X. Meng, P. Zhang, Z. Li, J. Xu, M.J. Reece, F. Gao, Dual-phase rare-earth-zirconate high-entropy ceramics with glass-like thermal conductivity, Journal of the European Ceramic Society 41(4) (2021) 2861-2869.
[29] W. Fan, Y. Bai, Y. Liu, T. Li, B. Li, L. Zhang, C. Gao, S. Shan, H. Han, Principal element design of pyrochlore-fluorite dual-phase medium- and high-entropy ceramics, Journal of Materials Science & Technology 107 (2022) 149-154.

[30] A.J. Wright, Q. Wang, S.-T. Ko, K.M. Chung, R. Chen, J. Luo, Size disorder as a descriptor for predicting reduced thermal conductivity in medium- and high-entropy pyrochlore oxides, Scripta Materialia 181 (2020) 76-81.
[31] B. Jiang, C.A. Bridges, R.R. Unocic, K.C. Pitike, V.R. Cooper, Y. Zhang, D.-Y. Lin, K. Page, Probing the Local Site Disorder and Distortion in Pyrochlore High-Entropy Oxides, Journal of the American Chemical Society 143(11) (2021) 4193-4204.
[32] D. Liu, Y. Wang, F. Zhou, B. Xu, B. Lv, A novel high-entropy (Sm0.2Eu0.2Tb0.2Dy0.2Lu0.2)2Zr2O7 ceramic aerogel with ultralow thermal conductivity, Ceramics International 47(21) (2021) 29960-29968.
[33] D. Zhang, Y. Yu, X. Feng, Z. Tian, R. Song, Thermal barrier coatings with high-entropy oxide as a top coat, Ceramics International 48(1) (2022) 1349-1359.
[34] D.R. Lowry, J.R. Boro, M. Blea-Kirby, N.R. Valdez, S.R. Bishop, Site Disorder as a Predictor for Compositionally Complex 5RE2Zr2O7 Ceramic Phase Stability, Journal of the American Ceramic Society 106(11) (2023) 7078-7087.
[35] F. Li, L. Zhou, J.-X. Liu, Y. Liang, G.-J. Zhang, High-entropy pyrochlores with low thermal conductivity for thermal barrier coating materials, Journal of Advanced Ceramics 8(4) (2019) 576-582.
[36] F. Vayer, C. Decorse, D. Bérardan, N. Dragoe, New entropy-stabilized oxide with pyrochlore structure: Dy2(Ti0.2Zr0.2Hf0.2Ge0.2Sn0.2)2O7, Journal of Alloys and Compounds 883 (2021) 160773.
[37] K. Ren, Q. Wang, G. Shao, X. Zhao, Y. Wang, Multicomponent high-entropy zirconates with comprehensive properties for advanced thermal barrier coating, Scripta Materialia 178 (2020) 382-386.
[38] K. Zhang, W. Li, J. Zeng, T. Deng, B. Luo, H. Zhang, X. Huang, Preparation of (La0.2Nd0.2Sm0.2Gd0.2Yb0.2)2Zr2O7 high-entropy transparent ceramic using combustion synthesized nanopowder, Journal of Alloys and Compounds 817 (2020) 153328.
[39] L. Zhou, F. Li, J.-X. Liu, Q. Hu, W. Bao, Y. Wu, X. Cao, F. Xu, G.-J. Zhang, High-entropy thermal barrier coating of rare-earth zirconate: A case study on (La0.2Nd0.2Sm0.2Eu0.2Gd0.2)2Zr2O7 prepared by atmospheric plasma spraying, Journal of the European Ceramic Society 40(15) (2020) 5731-5739.
[40] Z. Teng, L. Zhu, Y. Tan, S. Zeng, Y. Xia, Y. Wang, H. Zhang, Synthesis and structures of high-entropy pyrochlore oxides, Journal of the European Ceramic Society 40(4) (2020) 1639-1643.
[41] Z. Teng, Y. Tan, S. Zeng, Y. Meng, C. Chen, X. Han, H. Zhang, Preparation and phase evolution of high-entropy oxides A2B2O7 with multiple elements at A and B sites, Journal of the European Ceramic Society 41(6) (2021) 3614-3620.
